# Different clinical presentations of two renal transplant recipients with coronavirus disease 2019: a case report

**DOI:** 10.1186/s12879-020-05434-4

**Published:** 2020-09-25

**Authors:** Jing Li, Gang Chen, Mingmin Zhang, Shenghao Tu, Chao Chen

**Affiliations:** 1 Department of Integrated Traditional Chinese and Western Medicine, Tongji Hospital, Tongji Medical College, Huazhong University of Science and Technology, 1095 Jiefang Avenue, Wuhan, 430030 China; 2 Department of Orthopaedics, Union Hospital, Tongji Medical College, Huazhong University of Science and Technology, 1277 Jiefang Avenue, Wuhan, 430022 China

**Keywords:** Coronavirus disease 2019, Acute respiratory syndrome coronavirus 2, Renal transplant recipients, Immunosuppressive therapy, Case report

## Abstract

**Background:**

The Coronavirus Disease 2019 (COVID-19) caused by severe acute respiratory syndrome Coronavirus-2 has spread rapidly worldwide and disease spread is currently increasing. Data on the clinical picture of transplant recipients and management of the anti-rejection immunosuppressive therapy on COVID-19 infection are lacking.

**Case presentation:**

We report two cases of COVID-19 infection in renal transplant recipients with variable clinical presentations. The first patient presented with mild respiratory symptoms and a stable clinical course. The second patient had more severe clinical characteristics and presented with severe pneumonia and multi-organ failure. Both patients received a combination therapy including antiviral treatment and reduced immunosuppression therapy and finally recovered.

**Conclusions:**

We report COVID-19 infection in two renal transplant recipients with a favorable outcome but different clinical courses, which may provide a reference value for treating such patients.

## Background

As of early-May 2020, Coronavirus Disease 2019 (COVID-19) caused by severe acute respiratory syndrome Coronavirus-2 (SARS-CoV-2) has expanded to include over 3, 884,000 confirmed cases and over 272, 000 deaths, involving 176 countries around the world. The COVID-19 outbreak is spreading worldwide and has become a major international concern. On March 11, the World Health Organization (WHO) upgraded the status of the COVID-19 outbreak from epidemic to pandemic. Patients with COVID-19 may develop severe symptoms of acute respiratory infection, particularly those with comorbid conditions tend to have a high mortality rate [1].

Thus far, there are no specific therapeutic agents for COVID-19, and supportive care is the mainstay of management strategies. Clinical trials evaluating potential therapies, including remdesivir and chloroquine, are being conducted. Lopinavir and ritonavir are limited in transplantation due to drug-drug interactions with calcineurin inhibitors [2], and also have been found useless according to a recent study [3].

To the best of our knowledge, reported case of transplant recipient with COVID-19 is rare [4]. Data on the clinical picture of transplant recipients and management of the anti-rejection immunosuppressive therapy on COVID-19 infection are lacking. We report two cases of COVID-19 in renal transplant recipients with variable clinical presentations.

## Case presentation

### Case 1

A 57-year-old man who underwent a living-related kidney transplant due to chronic glomerulonephritis in 2013, was admitted to Union hospital in Wuhan on February 11, 2020, complaining of an unexplained fever (up to a maximum of 39.2 °C) for 6 days. This was followed by cough, fatigue, nausea, and shortness of breath, while no chest pain, diarrhea, or abdominal pain were present. The patient had no history of smoking or alcohol abuse, cardiovascular disease, or pulmonary disease. His immunosuppressive regimen consisted of tacrolimus 1.5 mg orally twice daily (trough serum levels 5 ng/mL ~ 8 ng/mL during the past year), and mycophenolate mofetil 0.75 g twice daily until 2 days prior to his visit. And there was no acute rejection or augmented or adjusted on immunosuppression in recent 2 years prior to infection. Two days ago, the patient went to the outpatient center due to consistent fever and all his immunosuppressants were discontinued as the doctor advised.

**Fig. 1:**
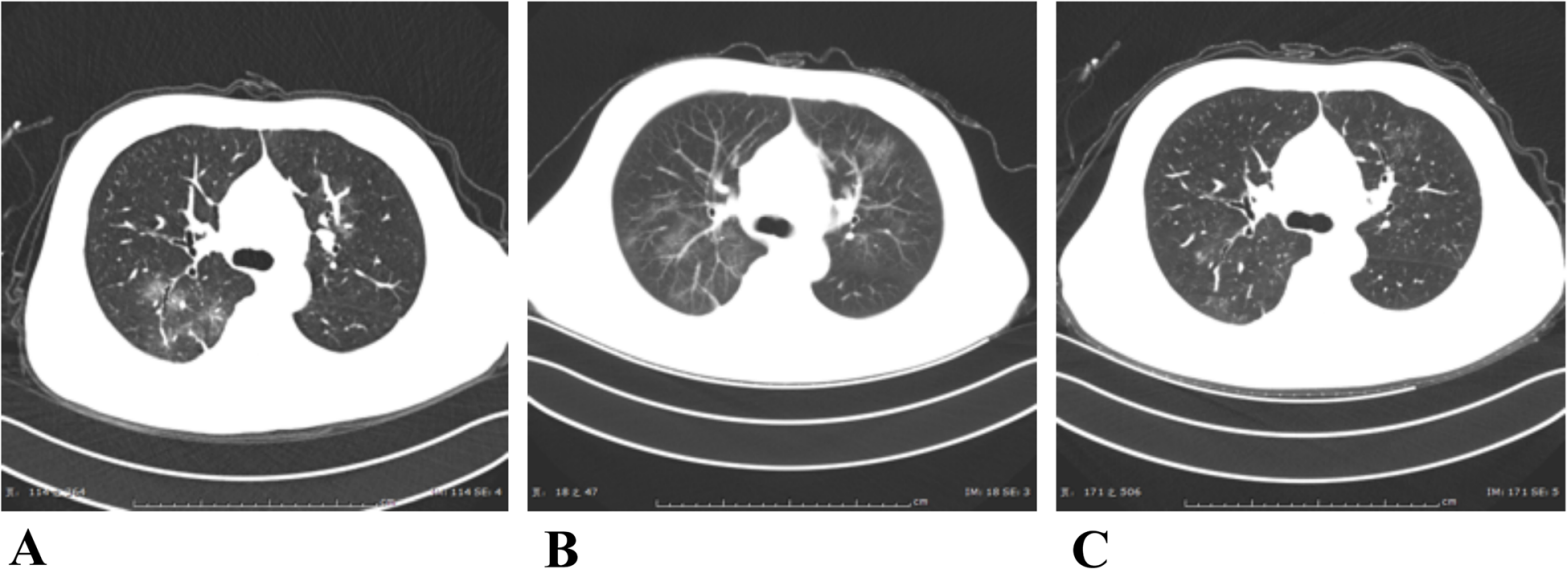
The chest computed tomography (CT) scan showed multiple patchy ground-glass opacities in superior lobe of bilateral lung and inferior lobar of left lung on admission (**a**), aggravation (increased area of patchy ground-glass opacities) on day 4 (**b**), and significant improvement of bilateral ground-glass opacities on day 9 (**c**)

On admission, his chest computed tomography (CT) scan showed multiple patchy ground-glass opacities in the bilateral lungs (Fig. 1a). Laboratory testing revealed an absolute lymphocyte count of 0.98 × 10^9^/L (normal range, 1.1–3.2 × 10^9^/L), serum creatinine 142 μmolL (normal range, 59–104 μmolL), and estimated glomerular filtration rate (eGFR) of 49.3 ml/min/1.73 m^2^ (normal range, > 90 ml/min/1.73 m^2^). A nasopharyngeal swab specimen was obtained and sent for detection of SARS-CoV-2 according to the CDC guidelines [5]. In brief, throat-swab specimens from the upper respiratory tract were obtained and maintained in a viral-transport medium. SARS-CoV-2 was confirmed by real-time reverse transcription polymerase chain reaction (RT-PCR) as previously reported [6]. Eight respiratory pathogens were tested using IgM and IgG antibody tests including respiratory syncytial virus, influenza virus A, influenza virus B, adenovirus, legionella pneumoniae, mycoplasma, parainfluenza, and chlamydia. These negative results indicated that no co-infection was present. The patient was diagnosed with COVID-19 according to the positive detection of SARS-CoV-2 and chest CT display.

Combination therapy was initiated with immunoglobin (200 mg/kg/day), methylprednisolone (40 mg daily), recombinant human interferon-alpha 2b (10 million IU daily), arbidol hydrochloride (0.6 g daily), and biapenem (0.6 g daily), for 12 days, in order to inhibit virus replication and implement empirical antibiotic treatment. Four days after admission, the alanine aminotransferase level increased to 76 U/L (normal range, 0–40 U/L), and by day 8 it had increased further to 93 U/L, which indicated hepatic damage. Glutathione was then initiated by 1.8 g intravenous injection daily for 9 days. Immunosuppression was resumed on day 2 after admission, including tacrolimus 1 mg twice daily, and mycophenolate mofetil 0.375 g twice daily (adjusted to 0.75 g twice daily on day 10). High-flow humidification oxygen administration was used to prevent acute hypoxic respiratory failure.

**Fig. 2:**
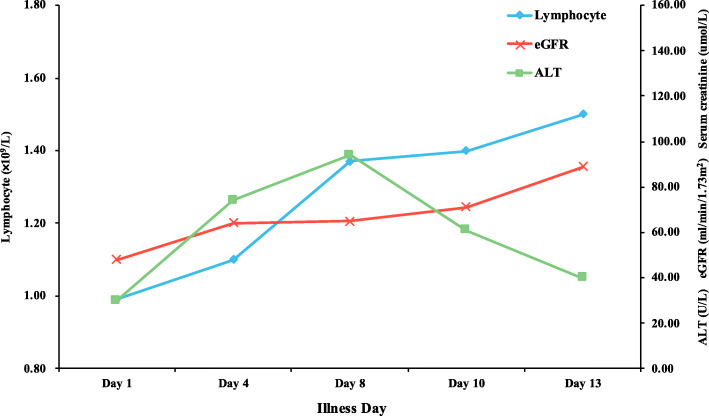
The lymphocyte count and estimated glomerular filtration rate (eGFR) is continuously increasing. The level of alanine aminotransferase (ALT) initially increased and then decreased during treatment

During treatment, the patient’s symptoms resolved with body temperature falling to between 36.3 °C and 37.1 °C, and his cough, nausea, and shortness of breath disappeared. The laboratory results were also improved, in particular, the lymphocyte count, serum creatinine, eGFR, and alanine aminotransferase (Fig. 2). And the kidney allograft function also improved during the course of therapy. On day 4, the second chest CT scan indicated that his pneumonia had aggravated (Fig. 1b). On day 9, the third chest CT scan (Fig. 1c) showed significant improvement of bilateral ground glass opacities compared to the previous scans. Based on the persistent negative results of SARS-CoV-2 RT-PCR on days 7 and 9, as well as the lung lesions partially decreased, the patient was discharged on day 13. To date, the patient has been in good health at home for 4 months.

### Case 2

A 55-year-old man, who underwent a renal transplant from a deceased donor in 2013 due to chronic kidney disease presented to the emergency department of Tongji Hospital on February 13, 2020, complaining of oliguria (< 400 ml) and a cough for 10 days, and shortness of breath for 2 days. He did not complain of fever, sore throat, or diarrhea. The patient had a history of surgery for urinary tract obstruction due to kidney stones and concomitant myocardial infarction in 2019. His immunosuppression was mycophenolate mofetil 0.5 g twice daily, tacrolimus 2.5 mg twice daily (trough serum levels 5 ng/mL ~ 10 ng/mL during the past year), and methylprednisolone 8 mg once daily. And there was no acute rejection or augmented or adjusted on immunosuppression in recent 6 months prior to infection.

**Fig. 3:**
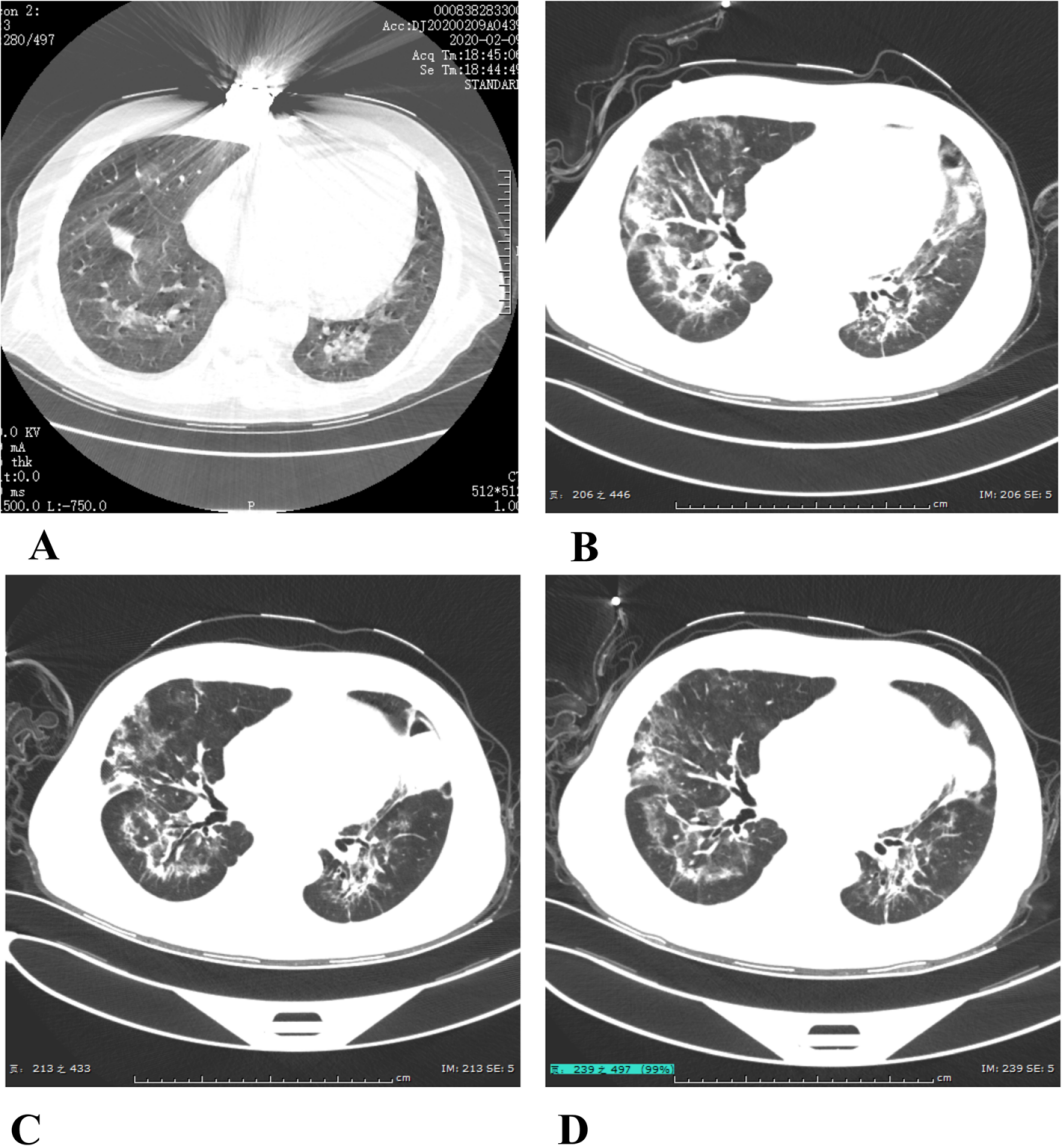
The chest computed tomography (CT) scan showed bilateral diffuse ground-glass changes and interstitial thickening on admission (**a**), aggravation (with large patchy opacities, local consolidation and pleural incrassation or conglutination) on day 10 (**b**), partial decrease of multiple patchy opacities on day 18 (**c**), and decrease of local consolidation on day 25 (**d**)

On admission, the patient received a 7 L/min of oxygen administration to maintain an oxygen saturation of 95%, with a blood pressure of 82/50 mmHg and a heart rate of 99 bpm. His chest CT scan showed bilateral diffuse ground-glass changes (Fig. 3a). Laboratory testing revealed an absolute lymphocyte count of 0.31 × 10^9^/L, serum creatinine 247 μmolL, eGFR 24.4 ml/min/1.73 m^2^, high-sensitivity troponin I (hsTNI) 312.8 pg/mL (normal range, < 34.2 pg/mL), and N-terminal pro-B-type natriuretic peptide (NT-proBNP) > 70,000 pg/mL (normal range, < 161 pg/mL). Nasopharyngeal swabs on admission were positive for SARS-CoV-2 RT-PCR. Then veno-venous hemodialysis and hemofiltration therapy was initiated. He developed arrhythmia (atrial fibrillation with rapid ventricular rate) on day 3 and promptly received synchronized cardioversion and noninvasive mechanical ventilation with bi-level positive airway pressure therapy. On day 5, the level of hsTNI increased to 1580.3 pg/mL and the NT-proBNP level was > 70,000 pg/mL, which indicated acute congestive heart failure. After diuresis, cardiotonics, steroids, and respiratory support, the patient’s clinical condition improved and he was transferred to the general ward for infectious diseases.

**Fig. 4:**
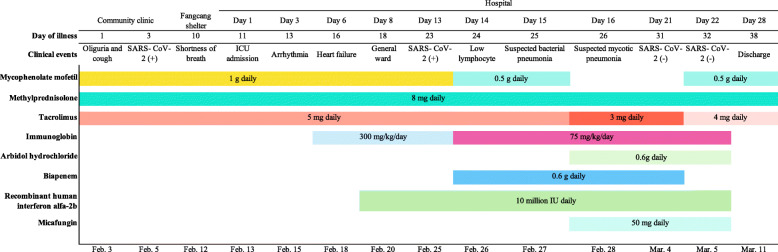
Medication strategy according to day of illness and day of hospitalization, February 3 to March 11, 2020

The patient received treatment with immunoglobin (300 mg/kg/day from day 6 to day 13 and adjusted to 75 mg/kg/day from day 14 to day 22), arbidol hydrochloride (0.6 g daily from day 16 to day 22), recombinant human interferon-alpha2 b (10 million IU daily from day 8 to day 22), and antimicrobial therapy consisting of biapenem (0.6 g daily from day 14 to day 21) and micafungin (50 mg daily from day 16 to day 22) according to the clinical events. Antimicrobial doses were adjusted to the patient’s creatinine clearance, and the immunosuppressive regimen was adjusted to the clinical condition, including CT scans, laboratory results, FK506 blood concentration, and symptoms (Fig. 4). On day 14, due to the persistent lymphocyte depletion and an abnormal ratio of lymphocyte subsets, the dose of immunoglobin was reduced to 75 mg/kg/day, and mycophenolate mofetil to 0.5 g daily. On day 16, mycophenolate mofetil was discontinued and tacrolimus was reduced to 3 mg daily due to the development of clinically suspected bacterial and mycotic pneumonia (invasive pulmonary aspergillosis according to the positive result of serum galactomannan test), and re-adjusted (mycophenolate mofetil 0.5 g daily and tacrolimus 4 mg daily) on day 22 due to the negative results of SARS-CoV-2 RT-PCR (Fig. 4).

**Fig. 5:**
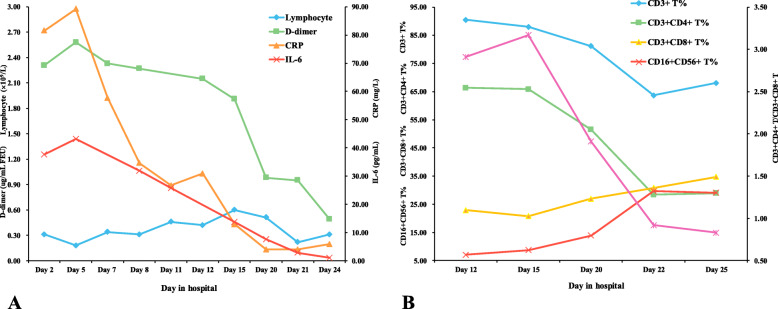
The levels of D-dimer, C-reactive protein (CRP), and interleukin 6 (IL-6) decreased, and the lymphocyte count fluctuated during treatment (**a**). The percentage of CD3^+^ T cells, CD3^+^CD4^+^ T cells, and the ratio of CD3^+^CD4^+^ T cells/CD3^+^CD8^+^ T cells decreased, and the percentage of CD3^+^CD8^+^ T cells and CD16^+^CD56^+^ T cells increased during treatment (**b**)

During treatment, the patient’s symptoms resolved and laboratory tests, including D-dimer, C-reactive protein (CRP), and interleukin 6 (IL-6), were improved significantly with days, although the lymphocyte count did not change significantly until the day of discharge (Fig. 5a). And the results of serum creatinine and eGFR were improved significantly, which indicated the kidney allograft function also improved. However, when comparing the lymphocyte subsets, the percentages of CD3^+^ T cells, CD3^+^CD4^+^ T cells (helper/inducer T cells), and the ratio of CD3^+^CD4^+^ T cells/CD3^+^CD8^+^ T cells decreased with the clinical course of the disease, while the percentage of CD3^+^CD8^+^ T cells (suppressor/cytotoxic T cells) and CD16^+^CD56^+^ T cells (natural killer cells, NK) increased with the clinical course of the disease (Fig. 5b). On day 10, the second chest CT scan indicated that his pneumonia aggravated with multiple patchy opacities and local consolidation (Fig. 3b). On day 18, the third chest CT scan showed significant improvement of multiple patchy opacities (Fig. 3c). On day 25, the fourth chest CT scan showed local consolidation decreased compared to the third scan (Fig. 3d). In addition, the high flow oxygen administration was removed on day 26. Given the remission of the disease, as well as the persistent negative results of SARS-CoV-2 RT-PCR, the patient was discharged on day 28. To date, the patient has been in good health at home for 3 months.

## Discussion and conclusions

Transplant recipients have a high susceptibility to viral pneumonia, such as severe acute respiratory syndrome (SARS), Middle East respiratory syndrome (MERS), and now COVID-19. Here, we report two cases of COVID-19 in renal transplant recipients with variable clinical presentations. Diagnosis was based on symptoms and chest CT scan, and was confirmed by real-time RT-PCR assays according to the Guidelines for the Diagnosis and Treatment of Novel Coronavirus (2019-nCoV) Infection (Trial Version 5) issued by the National Health Commission of the People’s Republic of China. The severity of COVID-19 is classified as asymptomatic, mild, severe, and critical cases of pneumonia (acute respiratory distress syndrome, sepsis, septic shock). In this report, the first patient was a mild case, and the second patient was a critical case.

In case 1, the patient had a stable clinical course. Although his pneumonia was aggravated on day 4 and an abnormal increase in serum creatinine and ALT indicated kidney and liver injury on day 8, his symptoms were relieved and the laboratory results improved after treatment, which may be associated with the continuous rise in lymphocyte count during treatment. We don’t know if the activation of lymphocytes is due to the withdrawal of immunosuppressive drugs for 3 days at the early stage or just be part of the natural course of the disease.

In case 2, the patient had more severe clinical characteristics, presenting with severe pneumonia and multi-organ failure. The patient’s heart failure was probably in part related to the excess fluid volume caused by his renal failure and myocardial infarction. Although there is currently no evidence to suggest that viral tropism to uroepithelial cells plays a role in causing acute renal failure in COVID-19, it has been confirmed as a cause of MERS CoV infection [7]. Secondary mixed bacterial-fungal pneumonia was highly suspected on day 16 after admission, and was accompanied by a positive result for SARS-CoV-2 RT-PCR. Then we reduced the dosage of anti-rejection medications. On day 22, the test was negative and the patient’s condition improved markedly, which indicated that reduction of immunosuppression may be helpful at that time.

In these two cases, we observed different profiles of lymphocyte counts, which fluctuated below the normal range in case 2 and continuously rose within the normal range in case 1. Similarly, previous studies have suggested that lymphocytopenia is a typical laboratory abnormality and considered disease severity related to highly pathogenic coronavirus infections [8, 9]. As the disease progressed and the clinical status deteriorated, the levels of lymphocytes progressively decreased, which may explain why the clinical course of the two patients was so different [10]. In addition, kinetic changes in lymphocyte subsets in case 2 showed that the percentage of CD3^+^CD4^+^ T cells decreased and the percentage of CD3^+^CD8^+^ T cells and NK cells increased as the disease improved. When CD4^+^ T cells are below 200 cells/mm^3^, patients with lymphocyte depletion have increased susceptibility to fungal infections [11], as was observed on day 16. Although dramatic loss of CD4^+^ and CD8^+^T cells in COVID-19 patients has been reported previously, it is unclear whether the increase in CD8^+^ T cells and NK cells were responsible for the recovery.

Inflammatory cytokine storm is another important characteristic in patients with severe COVID-19, which can rapidly cause severe immune damage to multiple organs and can ultimately be life-threatening. In line with this, we observed that the levels of D-dimer, CRP, and IL-6 were elevated initially and then decreased significantly during recovery, as evidenced by relieving pulmonary lesions on chest CT scan in case 2. It is also supported by a previous study that demonstrated that the decrease in IL-6 was closely related to treatment effectiveness, while an increase in IL-6 indicated disease exacerbation [12]. A cytokine storm is directly or indirectly caused by SARS-CoV-2 infection, which can activate pathogenic T cells and produce IL-6 and granulocyte-macrophage colony stimulating factor (GM-CSF). Together, these changes further activate CD14^+^CD16^+^ inflammatory monocytes and produce more IL-6 and other inflammatory factors [13]. We suggest that IL-6 might be a valuable candidate for monitoring severe type COVID-19.

Since literature on solid organ transplant recipients with COVID-19 is accumulating rapidly, there are currently several published case series and case reports, including kidney, liver, heart and lung transplant recipients. The majority of patients were male (82.5%), with an average age of 58 years and receiving maintenance immunosuppression that included tacrolimus with mycophenolate and steroids [14]. Recipients described were between 1 month and 22 years post-transplant. The most prominent symptoms were fever (58–90%) and cough (53–90%), lymphopenia (67–80%) and elevated CRP (49–100%) [15].

Although there are concerns that transplant recipients may have a more severe clinical course due to their immunosuppression and high rate of comorbid conditions. According to published reports, most of transplant recipients with COVID-19 presented with similar symptoms, laboratory values, imaging, and outcomes to those of immunocompetent patients [16–20]. Review of the clinical courses showed that most of the transplant patients were managed successfully without progression of the disease. However, some other studies revealed that transplant recipients experienced more severe COVID-19 pneumonia and a more difficult recovery than their infected family members and those in the general population [21–23]. In a case series of 12 patients with a history of kidney transplantation in Iran [24], the fatality rate reached 67%, and the author concluded the majority of the clinical features in deceased cases were similar to those from other adult studies for the general population.

Regarding treatment, the mainstay treatment for SARS-CoV-2 infection remains supportive care. In transplant recipient with long-term immunosuppression, a well balance is needed to overcome the initial viral response and the subsequent inflammatory response [25]. Although treatment of SARS-CoV-2 infection among transplant recipients varied by studies, decreasing immunosuppression and starting wide-spectrum antibiotics were the main therapy for most of the patients. According to previous studies [16, 18, 21, 23, 26],the majority of patients had received antimetabolite therapy (53–90%) (including remdesivir and lopinavir/ritonavir) and a smaller proportion had decreased or ceased calcineurin inhibitor (18–70%). Other therapies included hydroxychloroquine (86–91%), tocilizumab (6–16%), boosted protease inhibitors (50%) and immunoglobin (3–70%). Another review of 40 cases showed that mycophenolate was withdrawn in 90% of patients and calcineurin inhibitor in 72.2%. Steroids was maintained in 26 (81.3%) and started in 7 patients. There are 39 (97.5%) patients received antiviral therapy (including lopinavir, oseltamivir, arbidol, darunavir, interferon and nitazoxanide) and 32 (80%) patients received hydroxychloroquine [14]. Although most cases received immunosuppression reduction, others studies reported patients with immunosuppression maintained and recovered successfully [27–29].

There is still controversy about the role of corticosteroids for management of COVID-19 pneumonia. In MERS-CoV, corticosteroids was associated with prolonged viral clearance time. However, recent evidence suggested a decrease in mortality in patients with acute respiratory distress syndrome when using corticosteroids [30]. As corticosteroids suppress the overwhelming inflammation mediated by the hyperimmune response and decrease alveolar exudation [31], the use of low to moderate doses of corticosteroids (0.5–1 mg/kg/d for less than 7 days) in select patients has been recommended by the Chinese Thoracic Society [32]. Colchicine may also alleviate this cytokine storm through a distinct pathway. Gandolfini et al. [33] reported that the administration of colchicine may reduce the exaggerated inflammatory response in transplant recipients with COVID-19. It should be noted that whatever medical treatments are used, it is important to find a delicate balance that maximizes the benefits and minimizes the potential harm.

Due to the small number of patients, it remains unclear whether the natural history of COVID-19 is altered in transplant recipients. And also, it is difficult to make conclusions that whether the reduction of immunosuppressive regimen led to the activation of lymphocytes and thus played a part during the course of the disease.

We report COVID-19 infection in two renal transplant recipients with a favorable outcome but different clinical courses, which may provide a reference value for treating such patients.

## Data Availability

All material and data described in the manuscript are available upon request to the corresponding author of the present article.

## Abbreviations

ALT: Alanine aminotransferase
COVID-19: Coronavirus Disease 2019
CRP: C-reactive protein
CT: Computed tomography
eGFR: Estimated glomerular filtration rate
IL-6: Interleukin 6
RT-PCR: Reverse transcription polymerase chain reaction
SARS-CoV-2: Severe acute respiratory syndrome coronavirus 2
hsTNI: High-sensitive troponin I
NT- proBNP: N-terminal pro-B-type natriuretic peptide
NK: Natural killer cell
SARS: Severe acute respiratory syndrome
MERS: Middle East respiratory syndrome
GM-CSF: Granulocyte-macrophage colony stimulating factor

## References

[1] Guan WJ, Ni ZY, Hu Y, Liang WH, Ou CQ, He JX, et al. Clinical characteristics of coronavirus disease 2019 in China. N Engl J Med. 2020. 10.1056/NEJMoa2002032 [Epub ahead of print]. 10.1056/NEJMoa2002032 PMC7092819 32109013

[2] Michaels MG, La Hoz RM, Danziger-Isakov L, Blumberg EA, Kumar D, Green M, et al. Coronavirus disease 2019: Implications of emerging infections for transplantation. Am J Transplant. 2020. 10.1111/ajt.15832 [Epub ahead of print]. 10.1111/ajt.15832 PMC9800450 32090448

[3] Cao B, Wang Y, Wen D, Liu W, Wang J, Fan G, et al. A trial of Lopinavir-ritonavir in adults hospitalized with severe Covid-19. N Engl J Med. 2020. 10.1056/NEJMoa2001282 [Epub ahead of print]. 10.1056/NEJMoa2001282 PMC7121492 32187464

[4] Zhu L, Xu X, Ma K, Yang J, Guan H, Chen S, et al. Successful Recovery of COVID-19 Pneumonia in a Renal Transplant Recipient With Long-Term Immunosuppression. Am J Transplant. 2020;20(7):1859–63. 10.1111/ajt.15869. 10.1111/ajt.15869 PMC7228349 32181990

[5] Centers for Disease Control and Prevention. Interim Guidelines for Collecting, Handling, and Testing Clinical Specimens from Persons Under Investigation (PUIs) for Coronavirus Disease 2019 (COVID-19). Accessed 21 Mar 2020. (https://www.cdc.gov/coronavirus/2019-nCoV/guidelines-clinical-specimens.html).

[6] Corman V, Bleicker T, Brünink S, Drosten C, Landt O, Koopmans M, et al. Diagnostic detection of Wuhan coronavirus 2019 By real-time RT-PCR. Geneva: World Health Organization. . (https://www.who.int/docs/default-source/coronaviruse/wuhan-virus-assay-v1991527e5122341d99287a1b17c111902.pdf.

[7] Eckerle I, Müller MA, Kallies S, Gotthardt DN, Drosten C (2013). In-vitro renal epithelial cell infection reveals a viral kidney tropism as a potential mechanism for acute renal failure during Middle East respiratory syndrome (MERS) coronavirus infection. Virol J.

[8] Huang C, Wang Y, Li X, Ren L, Zhao J, Hu Y (2020). Clinical features of patients infected with 2019 novel coronavirus in Wuhan. China Lancet.

[9] Chen N, Zhou M, Dong X, Qu J, Gong F, Han Y (2020). Epidemiological and clinical characteristics of 99 cases of 2019 novel coronavirus pneumonia in Wuhan, China: a descriptive study. Lancet..

[10] Xu Z, Shi L, Wang Y, Zhang J, Huang L, Zhang C, et al. Pathological findings of COVID-19 associated with acute respiratory distress syndrome. Lancet Respir Med. 2020. 10.1016/S2213-2600(20)30076-X. 10.1016/S2213-2600(20)30076-X PMC7164771 32085846

[11] Cunningham-Rundles C, Ponda PP (2005). Molecular defects in T- and B-cell primary immunodeficiency diseases. Nat Rev Immunol.

[12] Rose-John S, Winthrop K, Calabrese L (2017). The role of IL-6 in host defence against infections: immunobiology and clinical implications. Nat Rev Rheumatol.

[13] Gupta KK, Khan MA, Singh SK (2020). Constitutive inflammatory cytokine storm: a major threat to human health. J Interf Cytokine Res.

[14] Machado DJB, Ianhez LE (2020). COVID-19 pneumonia in kidney transplant recipients-where we are?. Transpl Infect Dis.

[15] Johnson KM, Belfer JJ, Peterson GR, Boelkins MR, Dumkow LE (2020). Managing COVID-19 in renal transplant recipients: a review of recent literature and case supporting corticosteroid-sparing immunosuppression. Pharmacotherapy..

[16] Columbia University Kidney Transplant Program (2020). Early description of coronavirus 2019 disease in kidney transplant recipients in New York. J Am Soc Nephrol.

[17] Akalin E, Azzi Y, Bartash R, Seethamraju H, Parides M, Hemmige V (2020). Covid-19 and kidney transplantation. N Engl J Med.

[18] Pereira MR, Mohan S, Cohen DJ, Husain SA, Dube GK, Ratner LE (2020). COVID-19 in solid organ transplant recipients: initial report from the US epicenter. Am J Transplant.

[19] Ren ZL, Hu R, Wang ZW, Zhang M, Ruan YL, Wu ZY (2020). Epidemiologic and clinical characteristics of heart transplant recipients during the 2019 coronavirus outbreak in Wuhan, China: a descriptive survey report. J Heart Lung Transplant.

[20] Koczulla RA, Sczepanski B, Koteczki A, Kuhnert S, Hecker M, Askevold I, et al. SARS-CoV-2 infection in two patients following recent lung transplantation. Am J Transplant. 2020. 10.1111/ajt.15998 [Epub ahead of print]. 10.1111/ajt.15998 PMC7272871 32400084

[21] Zhu L, Gong N, Liu B, Lu X, Chen D, Chen S (2020). Coronavirus disease 2019 pneumonia in immunosuppressed renal transplant recipients: a summary of 10 confirmed cases in Wuhan. China Eur Urol.

[22] Latif F, Farr MA, Clerkin KJ, Habal MV, Takeda K, Naka Y (2020). Characteristics and outcomes of recipients of heart transplant with coronavirus disease 2019. JAMA Cardiol.

[23] Fernández-Ruiz M, Andrés A, Loinaz C, Delgado JF, López-Medrano F, San Juan R (2020). COVID-19 in solid organ transplant recipients: a single-center case series from Spain. Am J Transplant.

[24] Abrishami A, Samavat S, Behnam B, Arab-Ahmadi M, Nafar M, Sanei TM (2020). Clinical course, imaging features, and outcomes of COVID-19 in kidney transplant recipients. Eur Urol.

[25] Holzhauser L, Lourenco L, Sarswat N, Kim G, Chung B, Nguyen AB. Early experience of COVID-19 in 2 heart transplant recipients: Case reports and review of treatment options. Am J Transplant. 2020. 10.1111/ajt.15982 [Epub ahead of print]. 10.1111/ajt.15982 PMC7267352 32378314

[26] Fung M, Chiu CY, DeVoe C, Doernberg SB, Schwartz BS, Langelier C, et al. Clinical Outcomes and Serologic Response in Solid Organ Transplant Recipients with COVID-19: A Case Series from the United States. Am J Transplant. 2020. 10.1111/ajt.16079 [Epub ahead of print]. 10.1111/ajt.16079 PMC7300859 32476258

[27] Wang J, Li X, Cao G, Wu X, Wang Z, Yan T (2020). COVID-19 in a kidney transplant patient. Eur Urol.

[28] Bussalino E, De Maria A, Russo R, Paoletti E (2020). Immunosuppressive therapy maintenance in a kidney transplant recipient SARS-CoV-2 pneumonia: a case report. Am J Transplant.

[29] Seminari E, Colaneri M, Sambo M, Gallazzi I, Di Matteo A, Roda S (2020). SARS Cov-2 infection in a renal-transplanted patient: a case report. Am J Transplant.

[30] Wu C, Chen X, Cai Y, Xia J, Zhou X, Xu S (2020). Risk factors associated with acute respiratory distress syndrome and death in patients with coronavirus disease 2019 pneumonia in Wuhan, China. JAMA Intern Med.

[31] Lansbury LE, Rodrigo C, Leonardi-Bee J, Nguyen-Van-Tam J, Lim WS. Corticosteroids as adjunctive therapy in the treatment of influenza: an updated Cochrane systematic review and meta-analysis. Crit Care Med. 2019. 10.1097/CCM.0000000000004093 [Epub ahead of print]. 10.1097/CCM.0000000000004093 31939808

[32] Shang L, Zhao J, Hu Y, Du R, Cao B (2020). On the use of corticosteroids for 2019-nCoV pneumonia. Lancet..

[33] Gandolfini I, Delsante M, Fiaccadori E, Zaza G, Manenti L, Degli Antoni A (2020). COVID-19 in kidney transplant recipients. Am J Transplant.

